# Exploring Perfluoroalkyl and Polyfluoroalkyl Substance Presence and Potential Leaching from Reverse Osmosis Membranes: Implications for Drinking Water Treatment

**DOI:** 10.1021/acs.est.4c04743

**Published:** 2024-08-22

**Authors:** Mohammad Sadia, Thomas L. ter Laak, Emile R. Cornelissen, Annemarie P. van Wezel

**Affiliations:** † Institute for Biodiversity and Ecosystem Dynamics, University of Amsterdam, P.O. Box 94240, Amsterdam, GE 1090, The Netherlands; ‡ KWR Water Research Institute, P.O. Box 1072, Nieuwegein, BB 3430, The Netherlands; § Centre for Advanced Process Technology for Urban Resource Recovery (CAPTURE), Ghent University, Frieda Saeysstraat 1, Gent 9052, Belgium

**Keywords:** reverse osmosis, thin-film membrane, drinking water production, PFAS leaching, water treatment

## Abstract

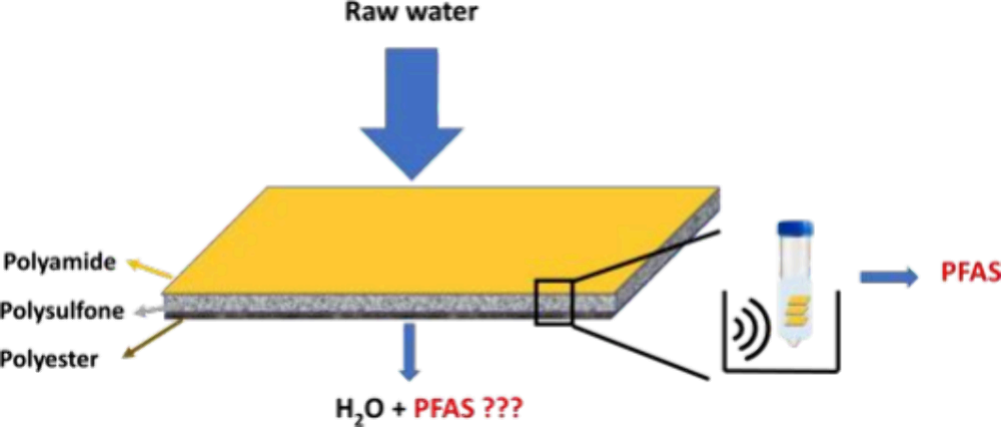

Reverse osmosis (RO) is increasingly used in drinking water production to effectively remove micropollutants, such as perfluoroalkyl and polyfluoroalkyl substances (PFAS). However, RO membranes themselves may contain PFAS, which can potentially leach into treated drinking water. Leaching experiments and direct total oxidizable precursor assays revealed the presence and leaching potential of PFOS (branched and linear), PFBA, PFHxA, PFNA, and PFOA in five selected commercial RO membranes. This resulted in the release of tens of milligrams of ΣPFAS per membrane element used in drinking water production. Depending on assumptions made regarding leaching kinetics and volume of produced water per membrane element, predicted concentrations of ΣPFAS in the produced water ranged from less than one up to hundreds of pg/L. These concentrations are two to four orders of magnitude lower than those currently observed in Dutch drinking waters. The origin of PFAS in the membranes remains unclear. Further research is needed to bridge the gap between the laboratory conditions as used in this study and the real-world conditions and for a full understanding of potential leaching scenarios. Such an understanding is critical for water producers using RO technologies to proactively manage and mitigate potential PFAS contamination.

## Abstract 2

The presence of PFAS in commercial reverse osmosis membranes has been confirmed, but further research is needed to assess their potential leaching into drinking water during full-scale production.

## 1 Introduction

Developments in water technology as applied during drinking water production have contributed importantly to the protection of human health. The presence of micropollutants (MPs) and their transformation products in drinking water sources received attention as an emerging global challenge toward the latter part of the previous century.^1−3^ To address this challenge, drinking water utilities are increasingly adopting advanced treatment technologies,^4^ such as sorption to activated carbon, oxidation by ozonation or ultraviolet light, and size separation by membrane treatment,^5^ to effectively remove undesired chemicals and produce safe and clean drinking water.

Reverse osmosis (RO) has emerged as an effective technology for removing many MPs in drinking water treatment. Nowadays, polyamide (PA) thin-film composite (TFC) membranes are the most commonly applied commercial RO membranes for the production of high-quality water.^6^ The RO membrane itself consists of three layers: a top layer (PA); a porous middle layer (poly(ether sulfone) (PES) or polysulfone (PS)); and a bottom layer (a nonwoven fabric support sheet of polyester (PE)).

The top layer is responsible for perm-selectivity and is usually formed by interfacial polymerization (IP) at the interface between two immiscible solutions: an aqueous solution containing a diamine, such as *m*-phenylenediamine, and a nonpolar organic solution containing a triacyl, such as trimesoyl chloride. This process results in the formation of an ultrathin selective PA layer on the PS support layer. The support layer provides mechanical strength during operational processes and is usually fabricated using the phase inversion method. This involves dissolving the polymer in a solvent to form a casting solution, followed by achieving phase separation using a certain physical technology.^7^ After synthesis, the PA-TFC membranes are stored in a solution (deionized water or sodium bisulfite solution) until use. The same applies to the support layer, which is stored to prevent pore collapsing with a storage solution until it is used for the IP process.^8^

The performance of PA-TFC membrane can be improved through optimization of the PA layer, achieved by tuning monomer ratio and concentration, reaction temperature, and reaction time, and incorporating additives such as organic and inorganic chemicals, surfactants, cosolvents, and ionic liquids.^9−15^ Additionally, optimizing the pore structure and the hydrophilicity of the support layer of TFC membranes can further improve the membrane performance. This optimization involves tuning factors such as polymer concentration, solvent composition, processing temperature, and the use of additives such as polyethylene glycol and polyvinylpyrrolidone.^16−19^ These optimization techniques have been successfully applied to both the PA and the support layers in commercial membranes, resulting in improved performance of PA-TFC membranes.^20^

Recently, the leaching of dissolved organic carbon from commercial RO membranes during the RO process has been confirmed, indicating the release of monomers used in the IP process.^21^ However, to the authors’ knowledge, studies concerning the leaching of additives used in membrane production are currently still lacking.

Per- and polyfluoroalkyl substances (PFAS) are a group of synthetic chemicals known for their advantageous physicochemical properties, including their surfactant behavior, heat resistance, and fat and water repellency, making them valuable for many industrial applications.

While the explicit use of PFAS in the fabrication of TFC membranes has not been reported by industry, fluoropolymer-based polymerization reactions including additives are commercially applied for TFC membrane production.^22^ They are approved in Europe as a processing additive on plastic materials and articles intended to come into contact with food by Commission Regulation (EU) 10/2011 of 14 January 14, 2011, and reported to be used in industries such as coating of equipment in chemical processing industry such as ducts, reactors, impellers, tanks, pipes, and fasteners.^23^ Additionally, the use of surfactants as additives can improve the IP process by aiding monomers in moving from the amine phase into the organic phase.^24,25,12^ Their synergistic use enhances membrane permeance and selectivity in solvent environments during TFC membrane synthesis.^26,27^ At the same time, RO membranes have been proven to relatively effectively remove long and short-chain PFAS.^28−30^

PFAS are recognized for their high persistence, accumulation potential, and associated hazards, leading to regulations of their occurrence in human relevant exposure media across different regions,^31−35^ although still, many data gaps in our knowledge on the occurrence and (eco)toxicity exist for the broader set of PFAS.^36^ Despite these efforts, elevated levels of PFAS continue to be found in environmental media.^37−39,30,40^ Current efforts are being made to reduce this emission by proposing a ban on the production, use, sale, and import of all PFAS in the EU while exempting PFAS used as a pesticide and pharmaceutical and proposed derogations.^41^

The potential emission of PFAS during water treatment due to leaching from the RO membrane materials, whether unintentionally introduced or intentionally added during membrane fabrication, has not been studied previously. This study aims to investigate PFAS residues in five commercially available RO membrane filters using leaching experiments, direct total oxidation precursors assay (TOPA), and analysis using a high-resolution mass spectrometer. The primary objective is to evaluate the presence of PFAS in RO membranes used for water purification and to quantify potential PFAS leaching during RO application based on assumptions regarding leaching kinetics.

## 2 Method and Material

### 2.1 Standards and Materials

Native and isotopic mass labeled standards (Table S1) were purchased from Wellington Laboratories (Guelph, Canada), excluding *n*-deuteriomethylperfluoro-1-*n*-octanesulfonamidoacetic acid-*d*_3_ (*N*-MeFOSAA-*d*_3_, > 99%) and *n*-ethylperfluoro-1-*n*-octanesulfonamidoacetic acid-*d*_5_ (*N*-EtFOSAA-*d*_5_, > 99%) which were purchased from Chiron (Trondheim, Norway), perfluoropropanoic acid (PFPrA, > 97%) from Sigma-Aldrich (Darmstadt, Germany), perfluoroethanesulfonic acid (PFEtS, > 98%) from Kanto Chemical (Japan), and *n*-methylperfluorobutanesulfonamide (MeFBSA, > 97%) from Apollo Scientific (Manchester, United Kingdom). A standard solution containing 45 PFAS analytes was made at 0.15 ng/μL in methanol, combining the mixture carboxylates (C3–C14), sulfonates (C3–C10; linear and branched), and a variety of precursors (C4–C12).

Additionally, LC-MS grade methanol and acetonitrile were purchased from Biosolve Chimie (Dieuze, France), while ammonium acetate (≥99%), hydrochloric acid (33%), and glacial acetic acid (≥99%) were obtained from Sigma-Aldrich. The ammonia solution (25%, analytical reagent grade) was sourced from Fisher Scientific (Massachusetts, United States).

### 2.2 Membrane Selection

Five different thin film composite commercial RO membranes, frequently investigated and widely employed in the literature and drinking water production, were randomly selected.^21,42^ A comprehensive summary of the selected membranes and their corresponding material properties is provided in Table 1.

**Table 1:** Specifications of the Selected Commercially Available Membranes

			membrane type			
name	brand	membrane application	**active layer**	**interlayer**	**support layer**	salt rejection (%)
FilmTec BW30	DuPont	brackish water	polyamide	polysulfone	polyester	99.50
FilmTec SW30HRLE	DuPont	seawater	polyamide	polysulfone	polyester	99.80
Suez AG-100 H	Suez	brackish water	polyamide	not disclosed	not disclosed	99.65
CPA5-LD	Hydranautics	brackish water	polyamide	not disclosed	not disclosed	99.70
TM720D-400	Toray	brackish water	polyamide	not disclosed	not disclosed	99.20

The investigated spiral-wound membranes were manually opened. Following this, a few drops of Milli-Q water were applied to the membrane sheet and wiped with a paper tissue to remove residual salt of the storage solution before being cut into fragments measuring 1 cm^2^. All tools used for opening and cutting the membrane sheets were prewashed with methanol to prevent contamination.

### 2.3 Experimental Setup

PFAS presence in membranes and their leaching were assessed using two approaches. First, the membrane fragments were extracted with Milli-Q water to examine possible leaching from the membranes (Section 2.3.1). Second, the total oxidizable precursor assay (TOPA) was directly applied to the membrane fragments (Section 2.3.2).

The TOPA assay enables the indirect measurement of both known and unknown PFAS precursors by converting them into known measurable perfluoroalkyl carboxylic acids (PFCA) and perfluoroalkanesulfonic acids.^43^ The harsh oxidizing conditions by TOPA might lead to oxidation of the polymers in the membrane materials, potentially enhancing the extraction of PFAS from the polymers. While TOPA is not specifically designed for extracting substances from polymer materials, it has been used on various polymers, including artificial turfs and textiles to detect PFAS.^44−46^ The decision to employ direct TOPA was motivated by the objective of gaining a comprehensive understanding of the PFAS presence in the RO membrane, particularly on those PFAS that did not leach during the first leaching experiment. Those PFAS might encompass both extractable/leachable and nonextractable/nonleachable PFAS in the RO membrane sheet. Leaching to Milli-Q water was used instead of drinking water, as drinking water or any other water source potentially contains PFAS that would bias the results.

#### 2.3.1 Membrane Leaching Experiment

A total of 40 membrane fragments of 1 cm^2^ were placed into a 50 mL polypropylene falcon tube containing 35 mL of Milli-Q water (pH = 7). Each of the tested commercial membranes was assessed in separate tubes. Mass-labeled extraction standard solution (10 μL, 100 pg μL^–1^) in methanol was spiked into the tubes, which were then placed in the sonication bath for 48h. After sonication, the water samples were adjusted to pH 4 using acetic acid, followed by solid-phase extraction (SPE) using an Oasis weak anion exchange WAX SPE cartridge (3 mL, 60 mg, 30 μm; Waters Corporation Milford, USA). The cartridges were preconditioned by passing subsequently 3 mL each of 0.1% ammonium hydroxide in methanol, pure methanol, and Milli-Q water. Subsequent to sample loading, the cartridges underwent a wash with 3 mL of ammonium acetate buffer (pH 4), followed by vacuum drying for 1 h. PFAS was subsequently eluted using 3 mL of 0.1% ammonium hydroxide in methanol. The resulting extracts were evaporated under nitrogen to achieve a volume of 65 μL, followed by the addition of 175 μL of 0.05% acetic acid in water and 10 μL of mass-labeled injection standard solution (100 pg μL^–1^). The 250 μL extract underwent vortex-mixing and centrifugation (5 min at 4000 rpm) and was then transferred to an LC vial for further chemical analysis.

#### 2.3.2 Total Oxidizable Precursor Assay

The TOPA was carried out directly on the membrane samples, following the method outlined by Lauria et al.^47^ In short, for each investigated membrane, 20 membrane fragments of 1 cm^2^, were placed in a 50 mL falcon tube to which were added 30 mL of Milli-Q water, 0.48 g of potassium persulfate, and 0.456 mL of NaOH (10 M). The tubes were then placed in an oven at 85 °C for 16 h. After cooling, the samples were spiked with 10 μL of a mass-labeled extraction standard solution (100 pg μL^–1^), and their pH was adjusted to 4 using HCl (33%). Then, the water samples were extracted using SPE following the procedure described in Section 2.3.1.

### 2.4 Quantification and Quality Control

The chemical analysis was performed on a Nexera UHPLC system (Shimadzu, Kyoto, Japan) coupled to a Bruker maXis 4 G q-TOF-high-resolution mass spectrometer (HRMS) and equipped with an IB-ESI source. Mass spectra were recorded in negative mode with a range of 50–1000 *m*/*z* and a 2 Hz sampling rate. Aliquots of 5 μL were injected into an Acquity UPLC CSH C18 column (130 Å, 2.1 × 150 mm, and 1.7 μm). The mobile phase consisted of 0.05% acetic acid in water (A) and 0.05% acetic acid in acetonitrile (B); details on eluent gradient and chromatographic separation can be found elsewhere.^30^ Identification and confirmation of target compounds were achieved by accurate mass within a mass window of 2 ppm, retention time match (≤0.20 min) of analytes detected in samples with corresponding standards in calibration solution, and confirming the presence of at least one fragment ion. The list of all target analytes and their exact mass used for confirmation are shown in Table S1. For branched isomers of PFOS, branched isomer standards were used for quantification. No branched isomers were detected for other PFAS.

The sample extraction procedure was conducted in triplicate for both the leaching experiment and the TOPA experiment. The relative standard deviation of the triplicate analyses was calculated to assess data reproducibility (RSD% < 20% for all samples). In the leaching experiment, procedural blank (Milli-Q water) and quality control (Milli-Q water spiked with native standards) were simultaneously extracted in triplicate with the samples. For the TOPA experiment, the procedural blank (tube without membrane) and quality control (20 μL perfluorooctanesulfonamide (FOSA) 500 pg μL^–1^) were also oxidized and extracted in triplicate alongside the samples, and full oxidation of FOAS was confirmed. A quality control sample (Milli-Q water spiked with native standards) was used for the SPE after TOPA was extracted alongside with TOPA samples, Table S2.

For HRMS instrument quality control, methanol injections were conducted before and after standard injections to assess any contamination in the LC system. Internal mass calibration for each analysis was performed by infusing a 50 mM sodium acetate solution in a water:methanol mixture (1:1, v:v) at the beginning of the analysis (0.1–0.5 min). The limit of quantification LOQ was determined using average analyte concentration in the procedural blanks plus ten times the standard deviation. In case one of the targeted 45 PFAS was not detected in the procedural blank, the LOQ was defined as the lowest point in the calibration curve,^40^Table S2.

The procedure blank showed high and inconsistent contamination, particularly with PFBS, leading to its exclusion from further evaluation. A limited number of PFAS were detected in the blank samples; details are provided in Table S2. This table also includes information on the limit of quantification and recoveries for the quality control samples for each of the 45 target PFAS.

### 2.5 Data Analysis

To extrapolate the findings from the leaching experiments to the industrial context of RO operations, typical parameters of commercial RO systems were considered. These parameters include a membrane surface area of 40 m^2^, a membrane lifespan ranging from 8 to 12 years, and average water permeate flux rates of 20 L m^–2^ h^–1^. In reality, these parameters may vary depending on the type of feedwater and membrane dimensions.^48,49^

Furthermore, three different scenarios for the kinetics of PFAS leaching from the RO membrane during operation were proposed: (1) complete leaching occurring during the initial week of operation, (2) complete leaching taking place during the initial month of operation, and (3) continuous leaching throughout the entire lifespan of approximately 12 years of the membrane. Equilibrium was not assumed as the membranes are continuously exposed to new clean permeate water. The PFAS concentration in the permeate water was calculated by dividing the amount of PFAS released per element by the volume of permeate water produced at specific time intervals in each scenario, an example of calculation provided in the SI.

By examining these scenarios, we aim to better understand the potential long-term implications of PFAS leaching in industrial RO processes and the risk of contaminating permeate water with PFAS.

## 3 Results and Discussion

### 3.1 PFAS Presence in RO Membranes: Leaching and TOPA Experiments

**Figure 1:**
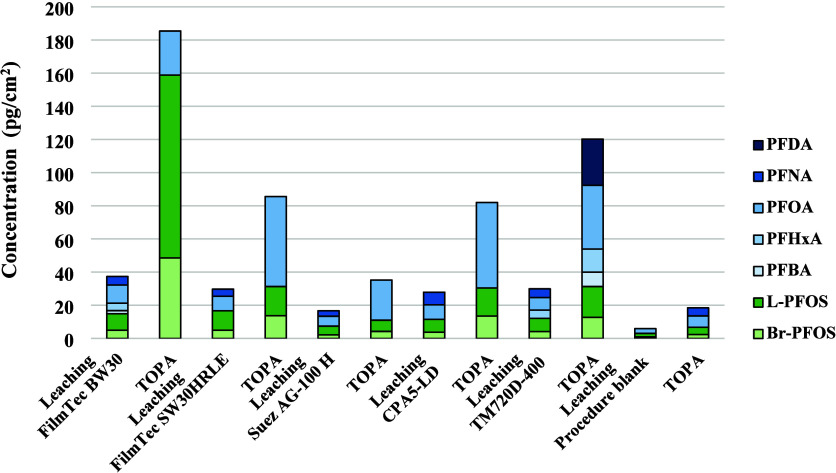
Concentrations (pg/cm^2^) of the detected PFAS in both leaching experiment and direct TOPA experiment for one cm^2^ of investigated RO membrane and in procedure blank.

Among the 45 investigated PFAS, 6 PFAS (specifically Br-/L-PFOS, PFBA, PFHxA, PFNA, and PFOA) were detected in the water from the leaching experiment with concentrations ranging from 17 to 38 pg/cm^2^ (Figure 1, Table S3). In the direct TOPA experiment, elevated concentrations of Br-/L-PFOS and PFOA were observed for all tested membranes (Figure 1, Table S3). Only the TM720D-400 membrane exhibited the detection of PFBA, PFDA, and PFHxA in the TOPA, while these three PFAS were not found in the TOPA extracts of the other membranes. PFNA was not detected in the direct TOPA experiment for any of the membranes, whereas it was detected in the leaching experiment. This might be attributed to the higher LOQ for PFNA in the TOPA experiment compared to the leaching experiment (Table S2).

The results for the different membranes are more variable for the TOPA compared to the data from leaching experiments. In general, TOPA resulted in higher PFAS loads extracted per cm^2^ than the water from the leaching experiment, Figure S1. This either suggests the presence of PFAS precursors that are oxidized into PFCA during TOPA, or PFAS that did not leach during the water leaching experiment but were mobilized by the TOPA treatment.^45^ Especially, the PFOS showed up to 16 times higher concentration per cm^2^ of membrane after TOPA treatment. The direct TOPA reported that high concentrations of oxidant can catalyze the hydrolysis of sulfonamides, leading to the formation of perfluoroalkyl sulfonates instead of carboxylates.^46^ Unlike standard TOPA, where radical oxidation by hydroxyl radicals primarily produces perfluoroalkyl carboxylates, the direct TOPA conditions favor the formation of perfluoroalkyl sulfonates, such as PFOS, due to the specific reactivity of sulfonamides under high oxidant concentrations.

Interestingly, in all tested membranes, only even-chain lengths (C4, C6, C8, and C10) were found after the TOPA, with no odd-numbered PFCA detected. This observation suggests that the PFAS precursors present are more likely to be sulfonamides rather than fluorotelomer compounds, as the fluorotelomer would result in chain-length shortening during the TOPA, producing odd-numbered PFCA.^50^ This finding aligns with studies on PFAS fingerprinting, which indicate specific precursor compounds leading to such patterns.^46^

The presence of PFAS observed in the membranes may be attributed to intentional or unintentional introduction during the manufacturing process of the membrane sheet by the incorporation of PFAS in the raw materials, such as monomers and solvents, or contamination during packaging or transportation. PFAS are also known to be used as polymer processing aids and might be used during the IP process.^51,52,23^ Additionally, contamination may be introduced unintentionally during the production where PFAS or fluoropolymer are known to be used in industry, such as using PFAS in lubricants and greases, cleaning solutions, and coating of industrial equipment.^23,52−54^ The dominant presence of the already globally banned PFOS and PFOA^55,56^ in all tested membranes suggests potential contamination, from the production environment or raw materials, which may originate from currently not banned PFAS precursors. The lack of public information on the use of PFAS during the production processes of the membranes in literature or industrial reports disables further confirmation of the source(s) of the PFAS presence in the membranes.

### 3.2 Implementations of PFAS Presence in the RO Membrane for Drinking Water Production

The identification of the PFAS presence in the RO membrane material, as confirmed in this study, prompts a critical consideration of the potential leaching behavior in real-world scenarios for applications of these membranes during drinking water production. While the experimental setup presented in this study provides valuable insights into the presence of PFAS within the composite membrane, both the leaching experiments and direct TOPA assays cannot fully simulate the complex dynamics of processes occurring during RO operation in practice.

The current leaching experiment, employing sonication as a harsh condition to induce PFAS leaching under controlled conditions, offers applicable insights for potential leaching. The sonication has a physical effect on membrane materials, by accelerating membrane aging and degrading the membrane surfaces, increasing pore density and porosity over time.^57^ This degradation process enhances PFAS extraction and release into the water phase.^58^ Thereby, it might reflect membrane aging over its lifetime. In the real-world application of RO, the membrane surface experiences different conditions influencing PFAS leaching. For example, the membrane is prewashed before operation to remove stabilizing agents for a period of one to a few hours, based on the technical manual of the producer for each membrane.^48^ This prewashing might lead to the removal of part of the PFAS before operation. Furthermore, the membrane during the RO operation process is exposed to an elevated pressure, varying temperatures, chemical cleaning agents, and biofilm formation on the membrane surface (fouling), all of which will contribute to membrane wear.

Furthermore, the 48 h sonication of membrane fragments extracted by Milli-Q water from both sides of the membrane does not fully replicate real-world conditions. In reality, the bottom side of the PA layer and the support layers (PS and PE) are exposed to the produced water, while the top side of the PA layer is exposed to the reject water. Therefore, leaching during RO operation in practice will differ from the 48 h sonication, as a disproportional part of the leached PFAS might actually end up in the reject water. Nevertheless, the sonication might provide an indication of the leaching potential.

Therefore, in this study, we can only preliminary indicate the scaling-up of the results from the PFAS leaching experiment conducted on a 40 cm^2^ membrane area to an industrial scale using commercial membranes. Thereby, as the worst realistic case, it was assumed that all PFAS in the RO membrane that leach do end up in the produced water, which potentially results in a release of ΣPFAS mass in the order of tens of milligrams per membrane element (first row in Table 2). The proposed scenarios did not consider PFAS removal during the prewashing steps before operation.

**Table 2:** Calculation of the Total Mass Release of ΣPFAS (pg) from One Element of Commercial RO (40 m^2^), and Predicted Concentrations (pg/L) of Permeate Water Following Different Kinetic Leaching Scenarios (Full Leaching in 1 week, 1 month, 12 years)

		FilmTec BW30		FilmTec SW30HRLE		Suez AG-100 H		CPA5-LD		TM720D-400	
		**leaching**	**TOPA**	**leaching**	**TOPA**	**leaching**	**TOPA**	**leaching**	**TOPA**	**leaching**	**TOPA**
mass release from RO element (pg ΣPFAS)		15 × 10^6^	74 × 10^6^	12 × 10^6^	34 × 10^6^	7 × 10^6^	14 × 10^6^	11 × 10^6^	33 × 10^6^	12 × 10^6^	48 × 10^6^
resulting concentrations (pg/L ΣPFAS)	1 week	111.6	550.6	89.3	253.0	52.1	104.2	81.8	245.5	89.3	357.1
	1 month	26.0	128.5	20.8	59.0	12.2	24.3	19.1	57.3	20.8	83.3
	12 years	0.2	0.9	0.1	0.4	0.1	0.2	0.1	0.4	0.1	0.6

The experimental setup used in this study does not elucidate the kinetics of PFAS leaching from the RO membranes during the production process. Extrapolating the results from the laboratory leaching and TOPA experiments to the context of common industrial RO operations, according to the suggested kinetic scenarios (Section 2.5), predicts a concentration range in the permeate water for the ΣPFAS originating from the membrane, varying between less than one to hundreds of pg/L (Table 2).

**Figure 2:**
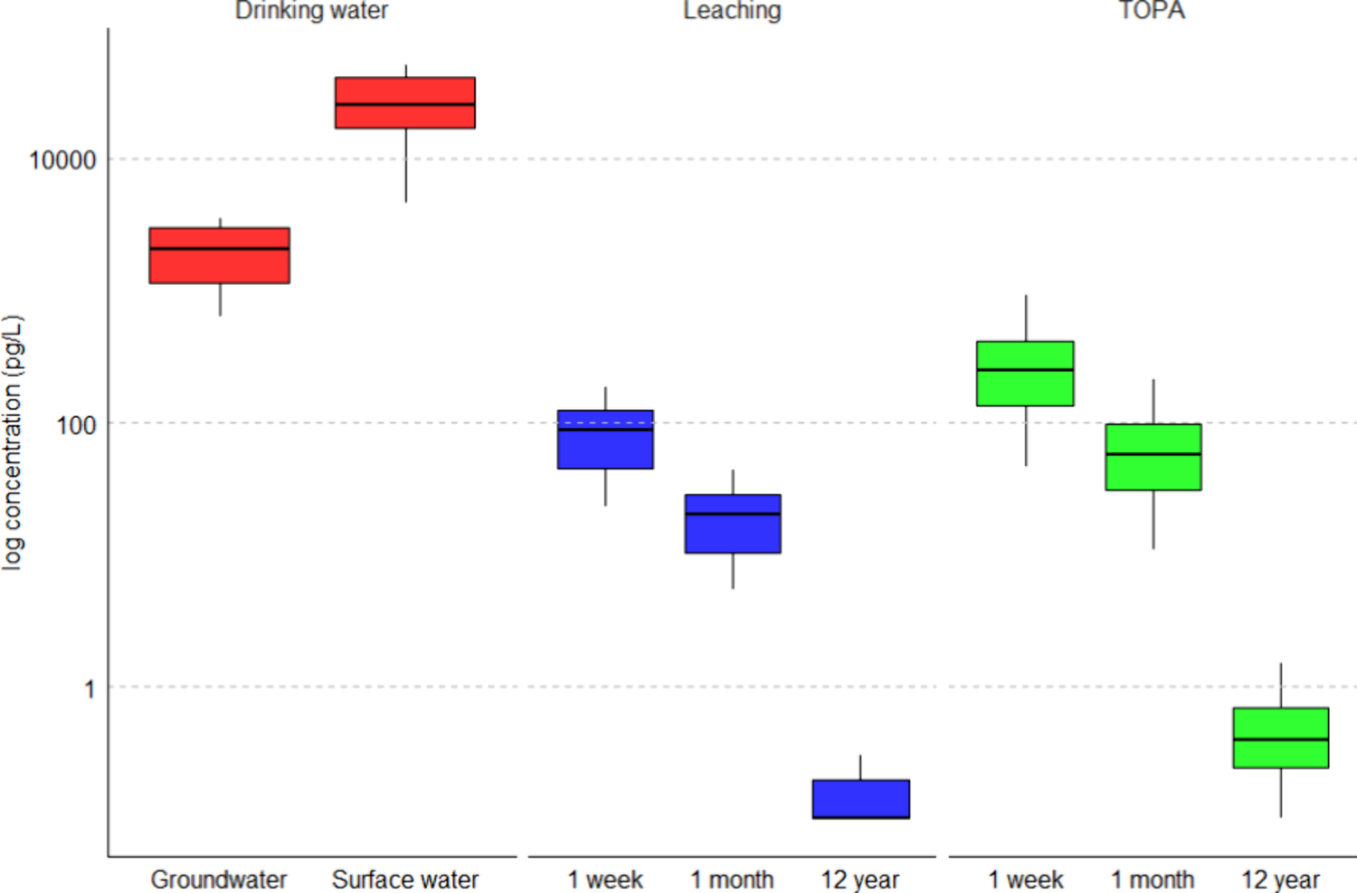
Box whisker plots present ΣPFAS concentrations (logarithm scale) in drinking water sourced from surface water and groundwater (in red, sum of only the seven PFAS detected in the tested membrane, Table S4),^30^ as compared to the predicted leaching kinetics scenarios (1 week, 1 month, 12 year) for the five tested membranes (blue for the leaching to water, green for direct TOPA). The whiskers represent the minimum and maximum concentrations, and the lower border of the box represents the first quartile (25%), the line inside the box the median, and the upper border is the third quartile (75%).

The concentrations of individual PFAS in both the month and year scenarios are currently undetectable using available analytical methods and fall well below the concentrations as observed in the drinking water produced from vulnerable groundwater or surface water in The Netherlands (Figure 2, Table S4), which are treated using different treatment processes such as sorption, oxidation, or RO filtration.^30^ Similarly, these concentrations are also well below those observed in drinking water produced outside The Netherlands.^59−61^ In the week scenario, the PFAS might be detectable, but resulting concentrations are still 2 orders of magnitude lower compared to concentrations in drinking water (Figure 2).^30^

The PFAS concentrations, both as individual and sum of group PFAS (Table S5), in the permeate water in all suggested kinetic scenarios comply with the existing guidelines for safe drinking water.^31−33,62^ However, in the first and second scenarios, they exceeded the recently restricted lifetime health advisory level (LHAL) proposed by US EPA,^35^ for both PFOS (EPA-LHDL 0.02 ng/L) and PFOA (EPA-LHDL 0.004 ng/L), with a few exceptions for PFOS in the second scenario (Table S5). It must be noted that membranes in practice are used much longer than a week to months. Nevertheless, these different scenarios for leaching kinetics highlight the need for a comprehensive understanding of the kinetics in practice.

While this study primarily focuses on the implications of PFAS presence and potential leaching in RO membranes for drinking water treatment, it is important to acknowledge that membranes are also widely used in various other industries, including the food industry.^63^ In these applications, membranes are utilized to prepare or treat aqueous matrices, presenting additional routes of exposure to PFAS. Therefore, the findings and considerations presented here are also relevant to other industries that utilize membrane technologies. Future research should take into account these broader applications and potential exposure routes to ensure comprehensive risk assessment and mitigation strategies.

The present study provides an initial step in understanding the presence of PFAS in the RO membranes and their potential for leaching. Further research is needed to understand the sources of PFAS within the membrane and to determine from which layer of the membrane the PFAS are leaching. Moreover, the kinetics of the leaching process needs further investigation under real-life conditions to assess the (temporal) variations in PFAS levels in permeate water and associated risks, particularly concerning critical windows during the development of unborn or newborns. Future research should aim to bridge these gaps between the current laboratory-scale experiments and full-scale industrial RO applications during drinking water treatment. Such research could assist the membrane-producing companies in providing protocols for proactive measures, such as adapting membrane conditioning and washing protocols before operation, to ensure safe application. Furthermore, this underscores the importance of ongoing research to prevent PFAS residues in drinking-water-contact materials, aligning with recent EU hygiene standards for materials and products that come into contact with drinking water, aimed at reducing such risks.

## Supporting Information Available

Calculation of ΣPFAS concentration in permeate water; leaching and TOPA experiment results (Figure S1); analytical standards and quality control data (Table S1,S2); PFAS concentrations in RO membrane and drinking water (Tables S3 and S4); predicted permeate concentrations (Table S5) (PDF)
