# Supplementary material for: Exploring Perfluoroalkyl and Polyfluoroalkyl Substance Presence and Potential Leaching from Reverse Osmosis Membranes: Implications for Drinking Water Treatment
Source: Environ Sci Technol. 2024 Aug 22;58(35):15799–806. doi: 10.1021/acs.est.4c04743 (PMC11375775; doi:10.1021/acs.est.4c04743)
Supplement: Supplementary file 1 — es4c04743_si_001.pdf [file es4c04743_si_001.pdf]

Supporting Information

Exploring PFAS presence and potential leaching from reverse osmosis membranes: Implications for drinking water treatment

Mohammad Sadia[a], Thomas L. ter Laak[a,b], Emile R. Cornelissen[b,c], Annemarie P. van Wezel[a]

[a] Institute for Biodiversity and Ecosystem Dynamics, University of Amsterdam, P.O. Box 94240, 1090 GE Amsterdam, The Netherlands.

[b] KWR Water Research Institute, P.O. Box 1072, 3430 BB, Nieuwegein, The Netherlands

[c] Centre for Advanced Process Technology for Urban Resource Recovery (CAPTURE), Ghent University, Frieda Saeysstraat 1, 9052 Gent, Belgium

Summary: 1 text, 1 figure, 5 tables

The following information is included

Content		Page
Text	Calculation of ΣPFAS concentration in permeate water	S2
Figure S1	Comparing the results from the leaching and TOPA experiments for each membrane	S3
Table S1	Information about the analytical standards.	S3
Table S2	Overview of quality control data.	S5
Table S3	Concentration of PFAS detected in the investigated RO membrane.	S6
Table S4	PFAS concentration in the drinking driven from previous publication	S7
Table S5	Predicted permeate water concentrations under different kinetic leaching scenarios.	S8

Calculation of ΣPFAS Concentration in Permeate Water

In Scenario 1, which assumes complete leaching of ΣPFAS occurring during the initial week of operation, the concentration of ΣPFAS in the permeate water was calculated as follows:

ΣPFAS released per element:

The total amount of ΣPFAS released from the membrane is calculated based on the measured concentration of ΣPFAS release from 1 cm^2 (Table S3).

For the leaching experiment:

ΣPFAS released per element = 37 $pg/cm^2 \times 40\ m^2 \times 10^4\ cm^2 = 15 \times 10^6$ pg.

Volume of permeate water produced for one element per scenario:

Using an average water permeate flux rate of 20 $L\ m^{-2}\ h^{-1}$, the volume of permeate water during the first week (168 h) for one element of 40 m^2 is calculated as:

Volume of permeate water = 20 $L\ m^{-2}\ h^{-1} \times 168\ h \times 40\ m^2$ = 134,400 L for one week. The volumes for one month (720 h) and 12 years (105192 h) were 576,000 L and 84,153,600 L respectively

ΣPFAS concentration in permeate water:

The concentration of ΣPFAS in the permeate water is calculated by dividing the total amount of ΣPFAS released (pg) by the volume of permeate water produced (L):

ΣPFAS concentration in one week = (15×10^6 pg) / 134,400 L = 111.6 pg/L.

The ΣPFAS concentration in one month and 12 years were 26.0 pg/L and 0.2 pg/L.

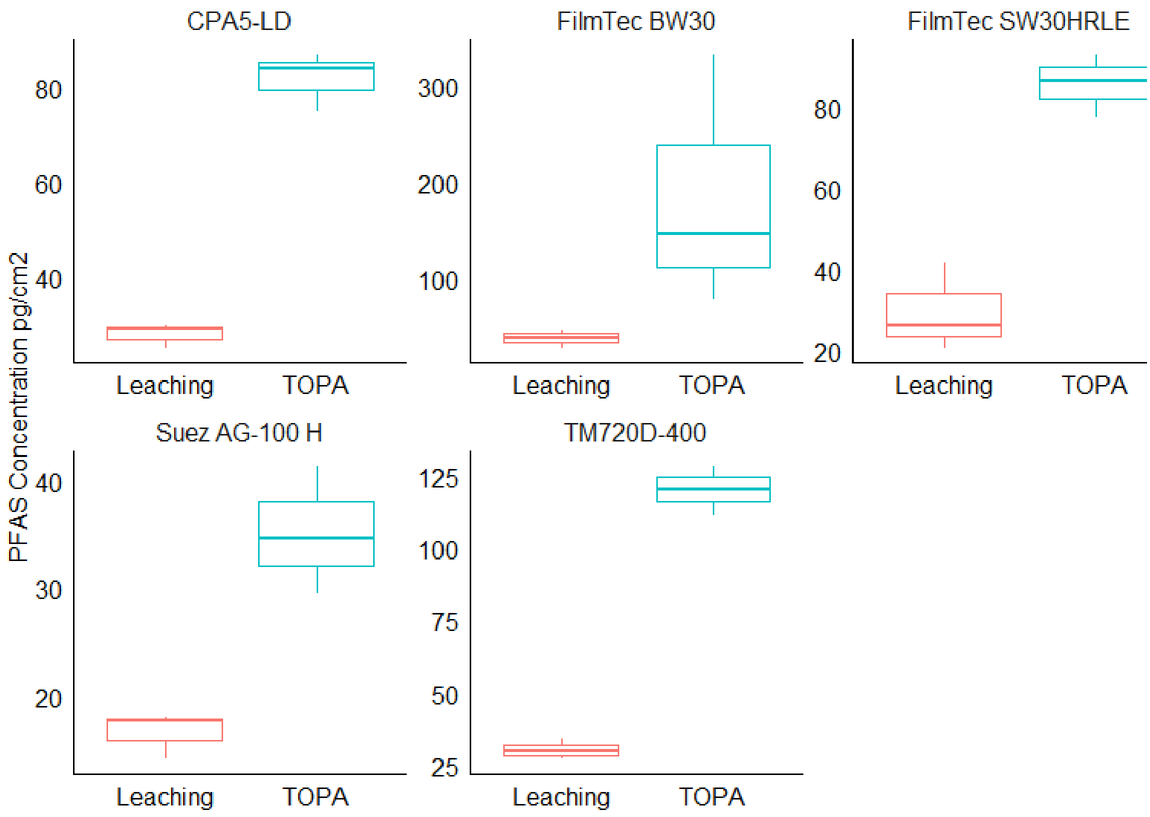

Figure S1. Boxplot showing the PFAS concentration (pg/cm²) for each membrane, comparing the results from the leaching and TOPA experiments.

Table S1. List of the compounds in the mixture standard of native, mass-labelled extraction and injection standards.

PFAS class	Compound	Acronym	Molecular Formula	Monoisotopic mass	Mass-labblele
perfluorocarboxylic acids	Pentafluoropropionic acid	PFPrA	$C_3HF_5O_2$	163.989670	M4PFBA
	Perfluorobutyric acid	PFBA	C4HF7O2	213.986476	M4PFBA
	Perfluoropentanoic acid	PFPeA	$C_5HF_9O_2$	263.983276	M5PFPeA
	Perfluorohexanoic acid	PFHxA	$C_6HF_{11}O_2$	313.980103	M5PFHxA
	Perfluoroheptanoic acid	PFHpA	$C_7HF_{13}O_2$	363.976898	M4PFHpA
	Perfluorooctanoic acid	PFOA	$C_8HF_{15}O_2$	413.973694	M8PFOA
	Perfluorononanoic acid	PFNA	$C_9HF_{17}O_2$	463.970520	M9PFNA
	Perfluorodecanoic acid	PFDA	$C_{10}HF_{19}O_2$	497.972412	M6PFDA
	Perfluoroundecanoic acid	PFUdA	$C_{11}HF_{21}O_2$	563.964111	M7PFUdA
	Perfluorododecanoic acid	PFDoA	$C_{12}HF_{23}O_2$	613.960938	MPFDoA
	Perfluorotridecanoic acid	PFTrDA	$C_{13}HF_{25}O_2$	663.957764	M7PFUdA
	Perfluorotetradecanoic acid	PFTeDA	$C_{14}HF_{27}O_2$	713.954529	M7PFUdA
perfluorosulfonic acids	Perfluoropropanesulfonic acid	PFPrS	$C_3HF_7O_3S$	249.953463	M3PFBS
	Potassium pentafluoroethanesulfonate	PFEtS	$C_2HF_5O_3S$	199.956650	M3PFBS
	Potassium perfluoro-1-butanesulfonate	PFBS	$C_4HF_9O_3S$	299..950256	M3PFBS
	Sodium perfluoro-1-pentanesulfonate	PFPeS	$C_5HF_{11}O_3S$	349.947083	M3PFBS
	Potassium perfluorohexanesulfonate	PFHxS	$C_6HF_{13}O_3S$	399.943878	M3PFHxS
	Sodium perfluoro-1-heptanesulfonate	PFHpS	$C_7HF_{15}O_3S$	449.940674	M3PFHxS
	Potassium perfluorooctanesulfonate	PFOS	$C_8HF_{17}O_3S$	499.937500	M8PFOS
	Sodium perfluoro-1-nonanesulfonate	PFNS	$C_9HF_{19}O_3S$	549.934326	M8PFOS
	Sodium perfluoro-1-decanesulfonate	PFDS	$C_{10}HF_{21}O_3S$	599.931091	M8PFOS
Precursors	Perfluorobutylsulphonamide	FBSA	$C_4H_2F_9NO_2S$	298.966248	M3PFBS
	Perfluorohexanesulfonamide	FHxSA	$C_6H_2F_{13}NO_2S$	398.959869	M3PFHxS
	Perfluorooctanesulfonamide	FOSA	$C_8H_2F_{17}NO_2S$	498.953491	M8PFOS
	N-methylperfluorooctane sulfonamidoacetic acid	N-MeFOSAA	$C_{11}H_6F_{17}NO_4S$	570.974609	d5-N-EtFOSAA
	N-ethylperfluorooctane sulfonamidoacetic acid	N-EtFOSAA	$C_{12}H_8F_{17}NO_4S$	584.990234	d5-N-EtFOSAA

	Sodium 1H. 1 H.2H.2H-perfluoro-1-hexanesulfonate	4:2FTS	$C_6H_5F_9O_3S$	327.981567	M2-4:2FTS
	Sodium 1 H. 1 H.2H.2H-perfluoro-1-octanesulfonate	6:2FTS	$C_8H_5F_{13}O_3S$	427.975189	M2-6:2FTS
	Sodium 1 H. 1 H.2H.2H-perfluoro-1-decanesulfonate	8:2FTS	$C_{10}H_5F_{17}O_3S$	527.968811	M2-8:2FTS
	Sodium dodecafluoro-3H-4.8-dioxanonanoate	ADONA	$C_7H_2F_{12}$O4	376.968322	M8PFOA
	Potassium 9-chlorohexadecafluoro-3-oxanonane-1-sulfonate	9Cl-PF3ONS	$C_8HClF_{16}O_4S$	531.902858	M8PFOA
	Potassium 11-chloroeicosafluoro-3-oxaundecane-1-sulfonate	11Cl-PF3OUDS	$C_{10}ClF_{20}HO_4S$	631.896471	M8PFOA
	Perfluoro-4-oxapentanoic acid	PF4OPeA	$C_4HF_7O_3$	229.981384	M4PFBA
	Perfluro-5-oxahexanoic acid	PF5OHxA	$C_5HF_9O_3$	279.978210	M3PFHxS
	Perfluoro-3.6-dioxaheptanoic acid	3.6-OPFHpA	$C_5HF_9O_4$	295.973114	M8PFOA
	Potassium perfluoro(2-ethoxyethane)sulfonate	PFEESA	$C_4F_9HO_4S$	315.945190	M3PFHxS
	Sodium 1 H. 1 H.2H.2H-perfluorododecanesulfonate	10:2FTS	$C_{12}H_5F_{21}O_3S$	627.962402	M2-8:2FTS
	Potassium perfluoro-4-ethylcyclohexanesulfonate	PFECHS	$C_8HF_{15}O_3S$	461..940674	M8PFOA
	3-Perfluoropropyl propanoic acid (3:3)	4:2FTA	$C_6H_5F_7O_2$	242.017776	M2-6:2FTA
	3-Perfluoropentyl propanoic acid (5:3)	6:2FTA	$C_8H_5F_{11}O_2$	342.011383	M2-6:2FTA
	3-Perfluoroheptyl propanoic acid (7:3)	8:2FTA	$C_{10}H_5F_{15}O_2$	442.005005	M2-8:2FTA
	N-ethylperfluoro-1-octanesulfonamidoacetic acid	N-EtFOSA	$C_{10}H_6F_{17}NO_2S$	526.984802	d9-N-EtFOSE
	N-methylperfluoro-1-octanesulfonamidoacetic acid	N-MeFOSA	$C_9H_4F_{17}NO_2S$	512..969116	d3-N-MeFOSA
	2-(N-ethylperfluoro-1-octanesulfonamido)-ethanol	N-EtFOSE	$C_{12}H_{10}F_{17}NO_3S$	571.010986	d3-N-MeFOSA
	2-(N-methylperfluoro-1-octanesulfonamido)-ethanol	N-MeFOSE	$C_{11}H_8F_{17}NO_3S$	556.995361	d9-N-EtFOSE
	N-(methyl) nonafluoro- butanesulfonamide	MeFBSA	$C_5H_4F_9NO_2S$	312.981903	M3PFBS
**Extraction standard**	Perfluoro-n-[$^{13}C_4$]butanoic acid	M4PFBA	13^$C_4HF_7O_2$	219.007173	
	Perfluoro-n-[13C5]pentanoic acid	M5PFPeA	13^$C_5HF_9O_2$	270.007334	
	Perfluoro-n-[1.2.3.4.6-13C5]hexanoic acid	M5PFHxA	13^$C_5CHF_{11}O_2$	320.004140	
	Perfluoro-n-[1.2.3.4-13C4]heptanoic acid	M4PFHpA	13^$C_4C_3HF_{13}O_2$	368.997592	
	Perfluoro-n-[13C8]octanoic acid	M8PFOA	13^$C_8HF_{15}O_2$	423.007818	
	Perfluoro-n-[13C9]nonanoic acid	M9PFNA	13^$C_9HF_{17}O_2$	474.007979	
	Perfluoro-n-[1.2.3.4.5.6-13C6]decanoic acid	M6PFDA	13^$C_6C_4HF_{19}O_2$	520.994721	
	Perfluoro-n-[1.2.3.4.5.6.7-13C7]undecanoic acid	M7PFUdA	13^$C_7C_4HF_{21}O_2$	571.994882	
	Perfluoro-n-[1.2-13C]dodecanoic acid	MPFDoA	13^$C_2C_{10}HF_{23}O_2$	616.974914	
	Sodium perfluoro-1-[2.3.4-13C3]butanesulfonate	M3PFBS	13^$C_3CHF_9O_3S$	303.967610	
	Sodium perfluoro-1-[1.2.3-13C3]hexanesulfonate	M3PFHxS	13^$C_3C_3H_2F_{13}O_3S$	404.969048	
	Sodium perfluoro-1-[13C8]octanesulfonate	M8PFOS	13^$C_8HF_{17}O_3S$	508.971610	
	Sodium 1 H. 1 H.2H.2H-perfluoro-1-[1.2-13C2]hexanesulfonate	M2-4:2FTS	13^$C_2C_4H_5F_9O_3S$	330.995555	
	Sodium 1 H. 1 H.2H.2H-perfluoro-1-[1.2-13C2]octanesulfonate	M2-6:2FTS	13^$C_2C_6H_5F_{13}O_3S$	430.989168	
	Sodium 1 H. 1 H.2H.2H-perfluoro-1-[1.2-13C2]decanesulfonate	M2-8:2FTS	13^$C_2C_8H_5F_{17}O_3S$	530.982781	
	2-Perfluorohexyl-[1.2-13C2]-ethanoic acid	M2-6:2FTA	13^$C_2C_6H_3F_{13}$O2	381.006532	
	2-Perfluorooctyl-[1.2-13C2]-ethanoic acid	M2-8:2FTA	13^$C_2C_8H_3F_{17}O_2$	481.000145	
	2-Perfluorodecyl-[1.2-13CJ-ethanoic acid	M2-10:2FTA	13^$C_2C_{10}H_3F_{21}O_2$	580.993758	
	Sodium bis(1 H. 1 H.2H.2H-[1.2-13C2]perfluorooctyl)phosphate	M4-6:2diPAP	13^$C_4C_{12}H_9F_{26}O_4P$	795.003025	
	Sodium bîs(1 H. 1 H.2H.2H-[1.2-13C2]perfluorodecyl)phosphate	M4-8:2diPAP	13^$C_4C_{16}H_9F_{34}O_4P$	994.990251	
	N-methyl-d3 -perfluoro-1-octanesulfonamide	d3-N-MeFOSA	2^$H_3C_9HF_{17}NO_2S$	516.995236	
	2-(N-ethyl-d5-perfluoro-1-octanesulfonamido)ethan-d4-ol	d9-N-EtFOSE	2^$H_9C_{12}HF_{17}NO_3S$	581.074761	
	N-Ethyl-n-perfluorooctanesulfonamidoacetic acld-d5	d5-N-EtFOSAA	2^$H_5C_{12}H_3F_{17}NO_4S$	591.028919	
**Injection standard**	Perfluoro-n-[2.3.4-13C3]butanoic acid	M3PFBA	13^$C_3CHF_7O_2$	218.003818	
	Perfluoro-n-(1.2-13C2]octanoic acid	M2PFOA	13^$C_2C_6HF_{15}O_2$	416.987689	
	Sodium perfluoro-1-[1.2.3.4-13C4]octanesulfonate	M4PFOS	13^$C_4C_4HF_{17}O_3S$	504.958190	
	N-ethyl-d5-perfluoro-1-octanesulfonamide	d5-N-EtFOSA-M	2^$H_5C_{10}HF_{17}NO_2S$	533.023439	
	2-(N-methyl-d3-perfluoro-1-octanesulfonamido)ethan-d4-ol	d7-N-MeFOSE-M	2^$H_7C_{11}HF_{17}NO_3S$	565.046558	
	N-Methyl-n-perfiuorooctanesu!fonamidoacetk acld-d3	N-MeFOSAA-d3	2^$H_3C_{11}H_3F_{17}NO_4S$	575.000715	

Table S2. Overview of quality control data, including average recoveries of quality control samples, average blank concentrations (pg), and the limit of quantification (LOQ) values in (pg). Standard deviations (SD) between triplicate samples are presented in brackets. The data presented the experimental data on the 40 cm^2 of membrane sheet.

PFAS name	Recovery (SD)	Leaching experiment		TOPA experiment	
		Blank (SD)	LOQ	Blank (SD)	LOQ
**PFPrA**	113% (27.05)	-	50	-	50
**PFPrS**	112% (22.73)	-	50	-	50
**PFEtS**	115% (25)	-	50	-	50
**PFBA**	99% (2.89)	-	50	-	50
**PFPeA**	114% (1.47)	-	50	-	50
**PFHxA**	108% (8.52)	-	50	-	50
**PFHpA**	95% (1.44)	-	50	-	50
**PFOA**	94% (15.81)	114 (3.75)	151	278 (34.70)	625
**PFNA**	100% (2.25)	-	50	192 (16.95)	362
**PFDA**	99% (0.91)	-	50	-	50
**PFUdA**	89% (25.41)	-	100	-	100
**PFDoA**	107% (0.7)	-	50	-	50
**PFTrDA**	168% (17.67)	-	100	-	100
**PFTeDA**	99% (40.09)	-	100	-	100
**PFBS**	-	-	-	-	-
**PFPeS**	113% (5.46)	-	50	-	50
**L-PFHxS**	90% (2.26)	-	50	-	50
**Br-PFHxS**	96% (2.98)	-	17	-	17
**PFHpS**	136% (6.87)	-	50	-	50
**L-PFOS**	123% (2.37)	83 (4.25)	126	174 (6.85)	250
**Br-PFOS**	107% (7.88)	38 (1.20)	50	92 (3.29)	125
**PFNS**	85% (12.84)	-	50	-	50
**PFDS**	56% (2.56)	-	50	-	50
**FBSA**	72% (0.86)	-	50	-	50
**FHxSA**	60% (1.78)	-	50	-	50
**FOSA**	43% (3.94)	-	50	-	50
**MeFOSAA**	85% (9)	-	50	-	50
**EtFOSAA**	77% (12)	-	50	-	50
**4:2FTS**	109% (0.38)	-	200	-	200
**6:2FTS**	115% (1.74)	-	200	-	200
**8:2FTS**	100% (3.89)	-	200	-	200
**ADONA**	102% (4.6)	-	50	-	50
**9Cl-PF3ONS**	54% (4.18)	-	50	-	50
**11Cl-PF3OUDS**	45% (1.88)	-	50	-	50
**PF4OPeA**	124% (4.02)	-	50	-	50
**PF5OHxA**	124% (0.6)	-	100	-	100
**3.6-OPFHpA**	123% (2.96)	-	200	-	200
**PFEESA**	107% (0.82)	-	50	-	50
**10:2FTS**	70% (3.55)	-	200	-	200
**PFECHS**	78% (0.6)	-	50	-	50
**3:3 FTCA**	95% (4.03)	-	200	-	200
**5:3 FTCA**	77% (3.04)	-	200	-	200
**7:3 FTCA**	65% (11.2)	-	200	-	200
**MeFBSA**	127% (4.15)	-	200	-	200

Table S3. Concentrations (pg/cm^2) of the detected PFAS in both leaching experiment and direct-TOPA experiment for 1 cm^2 of investigated RO membrane.

Membrane name	FilmTec BW30		FilmTec SW30HRLE		Suez AG-100 H		CPA5-LD		TM720D-400	
**Experiment**	**Leaching**	**TOPA**	**Leaching**	**TOPA**	**Leaching**	**TOPA**	**Leaching**	**TOPA**	**Leaching**	**TOPA**
**Br-PFOS**	5	49	5	14	2	4	4	13	4	13
**L-PFOS**	10	110	12	18	5	7	8	17	8	19
**PFBA**	2	< LOQ	< LOQ	< LOQ	< LOQ	< LOQ	< LOQ	< LOQ	< LOQ	9
**PFHxA**	4	< LOQ	< LOQ	< LOQ	< LOQ	< LOQ	< LOQ	< LOQ	5	14
**PFOA**	11	27	9	54	6	24	9	52	8	39
**PFNA**	5	< LOQ	4	< LOQ	3	< LOQ	8	< LOQ	5	< LOQ
**PFDA**	< LOQ	< LOQ	< LOQ	< LOQ	< LOQ	< LOQ	< LOQ	< LOQ	< LOQ	28
**∑PFAS**	37	185	30	86	17	35	28	82	30	120

Table S4. Concentrations (ng/L) of seven PFAS, detected in tested membrane, in the drinking water sourced from ground water and surface water and their treatment process[a], as imported from a previous study [1].

	**Surface water**											**Groundwater**						
	1	2	3	4	5	6	7	8	9	10	11	12	13	14	15	16	17	18
**Treatment process**	Advanced (PAC)	Advanced (Uv/PAC)	Advanced (PAC)	Advanced (PAC)	Advanced (Uv/GAC)	Advanced (Uv/GAC)	Advanced (GAC)	Advanced (GAC)	Advanced (Ozone/GAC)[b]	Advanced (RO/GAC)	Advanced (GAC)	Conventional	Conventional	Conventional	Conventional	Conventional	Conventional	Conventional
**PFBA**	10,18	13,41	8,05	10,82	4,35	3,60	1,27	12,28	3,49	4,46	7,90	0,43	0,40	0,31	0,13	0,30	1,17	0,97
**PFHxA**	8,80	7,60	6,85	7,61	4,62	3,34	0,30	3,03	0,75	1,23	0,68	<LOD	0,35	0,24	0,15	<LOD	<LOD	0,23
**L-PFOA**	3,63	2,23	6,68	7,17	3,19	2,52	0,74	4,02	4,28	1,60	1,07	0,06	0,87	0,61	0,34	0,10	0,28	0,35
**PFNA**	0,30	0,20	0,40	0,26	0,36	0,29	0,13	0,28	0,17	<LOD	<LOD	<LOD	0,09	0,08	<LOD	<LOD	<LOD	0,06
**PFDA**	0,17	0,05	0,30	0,15	0,18	0,14	0,18	0,14	<LOD	<LOD	<LOD	<LOD	0,08	0,28	0,15	<LOD	<LOD	0,13
**L-PFOS**	1,66	0,35	2,09	1,15	0,76	0,61	0,13	1,62	0,37	0,05	0,04	<LOD	0,12	0,08	0,09	0,04	0,06	0,05
**Br-PFOS**	1,17	0,46	2,22	1,64	0,93	0,75	0,20	1,86	0,50	0,10	0,03	<LOD	0,17	0,09	0,08	0,02	0,06	0,02
**∑PFAS**	25,91	24,30	26,59	28,80	14,39	11,25	2,95	23,23	9,56	7,44	9,72	0,49	2,08	1,69	0,94	0,46	1,57	1,81

[a] PAC: powder active carbon; GAC: granular active carbon; UV: ultraviolet; RO: reverse osmosis.
[b] The RO-permeate was mixed with raw water before conventional treatment.

Table S5. Predicted permeate water concentrations (pg/L) under different kinetic leaching scenarios (full leaching in 1 week, 1 month, 12 years). Bold values indicate concentrations exceeding the US EPA lifetime health advisory levels.

		FilmTec BW30		FilmTec SW30HRLE		Suez AG-100 H		CPA5-LD		TM720D-400	
		Leaching	TOPA	Leaching	TOPA	Leaching	TOPA	Leaching	TOPA	Leaching	TOPA
**1 week**	PFOS	**44,6**	**476,2**	**52,1**	**96,7**	**22,3**	**29,8**	**37,2**	**89,3**	**37,2**	**96,7**
	PFBA	7,4	-	-	-	-	-	-	-	-	22,3
	PFHxA	14,9	-	-	-	-	-	-	-	14,9	44,6
	PFOA	**29,8**	**81,8**	**29,8**	**163,7**	**14,9**	**74,4**	**22,3**	**156,3**	**22,3**	**111,6**
	PFNA	14,9	-	14,9	-	7,4	-	22,3	-	14,9	-
	PFDA	-	-	-	-	-	-	-	-	-	81,8
	∑PFAS	111,6	550,6	89,3	253	52,1	104,2	81,8	245,5	89,3	357,1
**1 month**	PFOS	10,4	**111,1**	12,2	**22,6**	5,2	6,9	8,7	20,8	8,7	**22,6**
	PFBA	1,7	-	-	-	-	-	-	-	-	5,2
	PFHxA	3,5	-	-	-	-	-	-	-	3,5	10,4
	PFOA	**6,9**	**19,1**	**6,9**	**38,2**	**3,5**	**17,4**	**5,2**	**36,5**	**5,2**	**26**
	PFNA	3,5	-	3,5	-	1,7	-	5,2	-	3,5	-
	PFDA	-	-	-	-	-	-	-	-	-	19,1
	∑PFAS	26	128,5	20,8	59	12,2	24,3	19,1	57,3	20,8	83,3
**12 year**	PFOS	0,1	0,8	0,1	0,2	0	0	0,1	0,1	0,1	0,2
	PFBA	0	-	-	-	-	-	-	-	-	0
	PFHxA	0	-	-	-	-	-	-	-	0	0,1
	PFOA	0	0,1	0	0,3	0	0,1	0	0,2	0	0,2
	PFNA	0	-	0	-	0	-	0	-	0	-
	PFDA	-	-	-	-	-	-	-	-	-	0,1
	∑PFAS	0,2	0,9	0,1	0,4	0,1	0,2	0,1	0,4	0,1	0,6

References:

(1) Sadia, M.; Nollen, I.; Helmus, R.; ter Laak, T. L.; Béen, F.; Praetorius, A.; van Wezel, A. P. Occurrence, Fate, and Related Health Risks of PFAS in Raw and Produced Drinking Water. *Environ. Sci. Technol.* **2023**, *57* (8), 3062–3074. https://doi.org/10.1021/acs.est.2c06015.
